# Evaluating “brain permeability”: A critical issue for the development of therapeutic agents for primary and metastatic brain tumors

**DOI:** 10.1093/neuonc/noag051

**Published:** 2026-03-12

**Authors:** Stuart A Grossman, Amy Barone, Roy E Strowd, Rana Rais, Elizabeth Duke, Emily Wearne, Lauren S L Price, Craig Hendrix, Karisa C Schreck, Glenn Lesser, Nader Sanai, Carlos Romo, Jana Portnow, Jaishri O Blakeley, David O Kamson, Burt Nabors, Peter Searson, Jann N Sarkaria, David Peereboom, David Schiff, Tatiana Prowell, William F Elmquist

**Affiliations:** Department of Oncology, Johns Hopkins University (S.A.G., R.R., C.H., K.C.S., C.R., J.O.B., D.O.K., P.S., T.P.); Food and Drug Administration (FDA), Silver Spring, Maryland, USA (A.B., E.D., E.W., L.S.L.P., T.P.); Department of Pharmaceutics, University of Minnesota, Minneapolis, Minnesota, USA (W.F.E.); Wake Forest School of Medicine, Winston-Salem, North Carolina, USA (R.E.S., G.L.); Department of Neurosurgery, Barrow’s Neurologic Institute, Phoenix, Arizona, USA (N.S.); Medical Oncology, City of Hope, Duarte, California, USA (J.P.); Department of Neurology, University of Alabama Birmingham, Birmingham, Alabama, USA (B.N.); Department of Radiation Oncology, Mayo Clinic, Rochester, Minnesota, USA (J.N.S.); Department of Medicine, Cleveland Clinic, Cleveland, Ohio, USA (D.P.); Department of Neurology, University of Virginia, Charlottesville, Virginia, USA (D.S.); Department of Oncology, Johns Hopkins University (S.A.G., R.R., C.H., K.C.S., C.R., J.O.B., D.O.K., P.S., T.P.); Food and Drug Administration (FDA), Silver Spring, Maryland, USA (A.B., E.D., E.W., L.S.L.P., T.P.); Department of Pharmaceutics, University of Minnesota, Minneapolis, Minnesota, USA (W.F.E.); Wake Forest School of Medicine, Winston-Salem, North Carolina, USA (R.E.S., G.L.); Department of Neurosurgery, Barrow’s Neurologic Institute, Phoenix, Arizona, USA (N.S.); Medical Oncology, City of Hope, Duarte, California, USA (J.P.); Department of Neurology, University of Alabama Birmingham, Birmingham, Alabama, USA (B.N.); Department of Radiation Oncology, Mayo Clinic, Rochester, Minnesota, USA (J.N.S.); Department of Medicine, Cleveland Clinic, Cleveland, Ohio, USA (D.P.); Department of Neurology, University of Virginia, Charlottesville, Virginia, USA (D.S.); Department of Oncology, Johns Hopkins University (S.A.G., R.R., C.H., K.C.S., C.R., J.O.B., D.O.K., P.S., T.P.); Food and Drug Administration (FDA), Silver Spring, Maryland, USA (A.B., E.D., E.W., L.S.L.P., T.P.); Department of Pharmaceutics, University of Minnesota, Minneapolis, Minnesota, USA (W.F.E.); Wake Forest School of Medicine, Winston-Salem, North Carolina, USA (R.E.S., G.L.); Department of Neurosurgery, Barrow’s Neurologic Institute, Phoenix, Arizona, USA (N.S.); Medical Oncology, City of Hope, Duarte, California, USA (J.P.); Department of Neurology, University of Alabama Birmingham, Birmingham, Alabama, USA (B.N.); Department of Radiation Oncology, Mayo Clinic, Rochester, Minnesota, USA (J.N.S.); Department of Medicine, Cleveland Clinic, Cleveland, Ohio, USA (D.P.); Department of Neurology, University of Virginia, Charlottesville, Virginia, USA (D.S.); Department of Oncology, Johns Hopkins University (S.A.G., R.R., C.H., K.C.S., C.R., J.O.B., D.O.K., P.S., T.P.); Food and Drug Administration (FDA), Silver Spring, Maryland, USA (A.B., E.D., E.W., L.S.L.P., T.P.); Department of Pharmaceutics, University of Minnesota, Minneapolis, Minnesota, USA (W.F.E.); Wake Forest School of Medicine, Winston-Salem, North Carolina, USA (R.E.S., G.L.); Department of Neurosurgery, Barrow’s Neurologic Institute, Phoenix, Arizona, USA (N.S.); Medical Oncology, City of Hope, Duarte, California, USA (J.P.); Department of Neurology, University of Alabama Birmingham, Birmingham, Alabama, USA (B.N.); Department of Radiation Oncology, Mayo Clinic, Rochester, Minnesota, USA (J.N.S.); Department of Medicine, Cleveland Clinic, Cleveland, Ohio, USA (D.P.); Department of Neurology, University of Virginia, Charlottesville, Virginia, USA (D.S.); Department of Oncology, Johns Hopkins University (S.A.G., R.R., C.H., K.C.S., C.R., J.O.B., D.O.K., P.S., T.P.); Food and Drug Administration (FDA), Silver Spring, Maryland, USA (A.B., E.D., E.W., L.S.L.P., T.P.); Department of Pharmaceutics, University of Minnesota, Minneapolis, Minnesota, USA (W.F.E.); Wake Forest School of Medicine, Winston-Salem, North Carolina, USA (R.E.S., G.L.); Department of Neurosurgery, Barrow’s Neurologic Institute, Phoenix, Arizona, USA (N.S.); Medical Oncology, City of Hope, Duarte, California, USA (J.P.); Department of Neurology, University of Alabama Birmingham, Birmingham, Alabama, USA (B.N.); Department of Radiation Oncology, Mayo Clinic, Rochester, Minnesota, USA (J.N.S.); Department of Medicine, Cleveland Clinic, Cleveland, Ohio, USA (D.P.); Department of Neurology, University of Virginia, Charlottesville, Virginia, USA (D.S.); Department of Oncology, Johns Hopkins University (S.A.G., R.R., C.H., K.C.S., C.R., J.O.B., D.O.K., P.S., T.P.); Food and Drug Administration (FDA), Silver Spring, Maryland, USA (A.B., E.D., E.W., L.S.L.P., T.P.); Department of Pharmaceutics, University of Minnesota, Minneapolis, Minnesota, USA (W.F.E.); Wake Forest School of Medicine, Winston-Salem, North Carolina, USA (R.E.S., G.L.); Department of Neurosurgery, Barrow’s Neurologic Institute, Phoenix, Arizona, USA (N.S.); Medical Oncology, City of Hope, Duarte, California, USA (J.P.); Department of Neurology, University of Alabama Birmingham, Birmingham, Alabama, USA (B.N.); Department of Radiation Oncology, Mayo Clinic, Rochester, Minnesota, USA (J.N.S.); Department of Medicine, Cleveland Clinic, Cleveland, Ohio, USA (D.P.); Department of Neurology, University of Virginia, Charlottesville, Virginia, USA (D.S.); Department of Oncology, Johns Hopkins University (S.A.G., R.R., C.H., K.C.S., C.R., J.O.B., D.O.K., P.S., T.P.); Food and Drug Administration (FDA), Silver Spring, Maryland, USA (A.B., E.D., E.W., L.S.L.P., T.P.); Department of Pharmaceutics, University of Minnesota, Minneapolis, Minnesota, USA (W.F.E.); Wake Forest School of Medicine, Winston-Salem, North Carolina, USA (R.E.S., G.L.); Department of Neurosurgery, Barrow’s Neurologic Institute, Phoenix, Arizona, USA (N.S.); Medical Oncology, City of Hope, Duarte, California, USA (J.P.); Department of Neurology, University of Alabama Birmingham, Birmingham, Alabama, USA (B.N.); Department of Radiation Oncology, Mayo Clinic, Rochester, Minnesota, USA (J.N.S.); Department of Medicine, Cleveland Clinic, Cleveland, Ohio, USA (D.P.); Department of Neurology, University of Virginia, Charlottesville, Virginia, USA (D.S.); Department of Oncology, Johns Hopkins University (S.A.G., R.R., C.H., K.C.S., C.R., J.O.B., D.O.K., P.S., T.P.); Food and Drug Administration (FDA), Silver Spring, Maryland, USA (A.B., E.D., E.W., L.S.L.P., T.P.); Department of Pharmaceutics, University of Minnesota, Minneapolis, Minnesota, USA (W.F.E.); Wake Forest School of Medicine, Winston-Salem, North Carolina, USA (R.E.S., G.L.); Department of Neurosurgery, Barrow’s Neurologic Institute, Phoenix, Arizona, USA (N.S.); Medical Oncology, City of Hope, Duarte, California, USA (J.P.); Department of Neurology, University of Alabama Birmingham, Birmingham, Alabama, USA (B.N.); Department of Radiation Oncology, Mayo Clinic, Rochester, Minnesota, USA (J.N.S.); Department of Medicine, Cleveland Clinic, Cleveland, Ohio, USA (D.P.); Department of Neurology, University of Virginia, Charlottesville, Virginia, USA (D.S.); Department of Oncology, Johns Hopkins University (S.A.G., R.R., C.H., K.C.S., C.R., J.O.B., D.O.K., P.S., T.P.); Food and Drug Administration (FDA), Silver Spring, Maryland, USA (A.B., E.D., E.W., L.S.L.P., T.P.); Department of Pharmaceutics, University of Minnesota, Minneapolis, Minnesota, USA (W.F.E.); Wake Forest School of Medicine, Winston-Salem, North Carolina, USA (R.E.S., G.L.); Department of Neurosurgery, Barrow’s Neurologic Institute, Phoenix, Arizona, USA (N.S.); Medical Oncology, City of Hope, Duarte, California, USA (J.P.); Department of Neurology, University of Alabama Birmingham, Birmingham, Alabama, USA (B.N.); Department of Radiation Oncology, Mayo Clinic, Rochester, Minnesota, USA (J.N.S.); Department of Medicine, Cleveland Clinic, Cleveland, Ohio, USA (D.P.); Department of Neurology, University of Virginia, Charlottesville, Virginia, USA (D.S.); Department of Oncology, Johns Hopkins University (S.A.G., R.R., C.H., K.C.S., C.R., J.O.B., D.O.K., P.S., T.P.); Food and Drug Administration (FDA), Silver Spring, Maryland, USA (A.B., E.D., E.W., L.S.L.P., T.P.); Department of Pharmaceutics, University of Minnesota, Minneapolis, Minnesota, USA (W.F.E.); Wake Forest School of Medicine, Winston-Salem, North Carolina, USA (R.E.S., G.L.); Department of Neurosurgery, Barrow’s Neurologic Institute, Phoenix, Arizona, USA (N.S.); Medical Oncology, City of Hope, Duarte, California, USA (J.P.); Department of Neurology, University of Alabama Birmingham, Birmingham, Alabama, USA (B.N.); Department of Radiation Oncology, Mayo Clinic, Rochester, Minnesota, USA (J.N.S.); Department of Medicine, Cleveland Clinic, Cleveland, Ohio, USA (D.P.); Department of Neurology, University of Virginia, Charlottesville, Virginia, USA (D.S.); Department of Oncology, Johns Hopkins University (S.A.G., R.R., C.H., K.C.S., C.R., J.O.B., D.O.K., P.S., T.P.); Food and Drug Administration (FDA), Silver Spring, Maryland, USA (A.B., E.D., E.W., L.S.L.P., T.P.); Department of Pharmaceutics, University of Minnesota, Minneapolis, Minnesota, USA (W.F.E.); Wake Forest School of Medicine, Winston-Salem, North Carolina, USA (R.E.S., G.L.); Department of Neurosurgery, Barrow’s Neurologic Institute, Phoenix, Arizona, USA (N.S.); Medical Oncology, City of Hope, Duarte, California, USA (J.P.); Department of Neurology, University of Alabama Birmingham, Birmingham, Alabama, USA (B.N.); Department of Radiation Oncology, Mayo Clinic, Rochester, Minnesota, USA (J.N.S.); Department of Medicine, Cleveland Clinic, Cleveland, Ohio, USA (D.P.); Department of Neurology, University of Virginia, Charlottesville, Virginia, USA (D.S.); Department of Oncology, Johns Hopkins University (S.A.G., R.R., C.H., K.C.S., C.R., J.O.B., D.O.K., P.S., T.P.); Food and Drug Administration (FDA), Silver Spring, Maryland, USA (A.B., E.D., E.W., L.S.L.P., T.P.); Department of Pharmaceutics, University of Minnesota, Minneapolis, Minnesota, USA (W.F.E.); Wake Forest School of Medicine, Winston-Salem, North Carolina, USA (R.E.S., G.L.); Department of Neurosurgery, Barrow’s Neurologic Institute, Phoenix, Arizona, USA (N.S.); Medical Oncology, City of Hope, Duarte, California, USA (J.P.); Department of Neurology, University of Alabama Birmingham, Birmingham, Alabama, USA (B.N.); Department of Radiation Oncology, Mayo Clinic, Rochester, Minnesota, USA (J.N.S.); Department of Medicine, Cleveland Clinic, Cleveland, Ohio, USA (D.P.); Department of Neurology, University of Virginia, Charlottesville, Virginia, USA (D.S.); Department of Oncology, Johns Hopkins University (S.A.G., R.R., C.H., K.C.S., C.R., J.O.B., D.O.K., P.S., T.P.); Food and Drug Administration (FDA), Silver Spring, Maryland, USA (A.B., E.D., E.W., L.S.L.P., T.P.); Department of Pharmaceutics, University of Minnesota, Minneapolis, Minnesota, USA (W.F.E.); Wake Forest School of Medicine, Winston-Salem, North Carolina, USA (R.E.S., G.L.); Department of Neurosurgery, Barrow’s Neurologic Institute, Phoenix, Arizona, USA (N.S.); Medical Oncology, City of Hope, Duarte, California, USA (J.P.); Department of Neurology, University of Alabama Birmingham, Birmingham, Alabama, USA (B.N.); Department of Radiation Oncology, Mayo Clinic, Rochester, Minnesota, USA (J.N.S.); Department of Medicine, Cleveland Clinic, Cleveland, Ohio, USA (D.P.); Department of Neurology, University of Virginia, Charlottesville, Virginia, USA (D.S.); Department of Oncology, Johns Hopkins University (S.A.G., R.R., C.H., K.C.S., C.R., J.O.B., D.O.K., P.S., T.P.); Food and Drug Administration (FDA), Silver Spring, Maryland, USA (A.B., E.D., E.W., L.S.L.P., T.P.); Department of Pharmaceutics, University of Minnesota, Minneapolis, Minnesota, USA (W.F.E.); Wake Forest School of Medicine, Winston-Salem, North Carolina, USA (R.E.S., G.L.); Department of Neurosurgery, Barrow’s Neurologic Institute, Phoenix, Arizona, USA (N.S.); Medical Oncology, City of Hope, Duarte, California, USA (J.P.); Department of Neurology, University of Alabama Birmingham, Birmingham, Alabama, USA (B.N.); Department of Radiation Oncology, Mayo Clinic, Rochester, Minnesota, USA (J.N.S.); Department of Medicine, Cleveland Clinic, Cleveland, Ohio, USA (D.P.); Department of Neurology, University of Virginia, Charlottesville, Virginia, USA (D.S.); Department of Oncology, Johns Hopkins University (S.A.G., R.R., C.H., K.C.S., C.R., J.O.B., D.O.K., P.S., T.P.); Food and Drug Administration (FDA), Silver Spring, Maryland, USA (A.B., E.D., E.W., L.S.L.P., T.P.); Department of Pharmaceutics, University of Minnesota, Minneapolis, Minnesota, USA (W.F.E.); Wake Forest School of Medicine, Winston-Salem, North Carolina, USA (R.E.S., G.L.); Department of Neurosurgery, Barrow’s Neurologic Institute, Phoenix, Arizona, USA (N.S.); Medical Oncology, City of Hope, Duarte, California, USA (J.P.); Department of Neurology, University of Alabama Birmingham, Birmingham, Alabama, USA (B.N.); Department of Radiation Oncology, Mayo Clinic, Rochester, Minnesota, USA (J.N.S.); Department of Medicine, Cleveland Clinic, Cleveland, Ohio, USA (D.P.); Department of Neurology, University of Virginia, Charlottesville, Virginia, USA (D.S.); Department of Oncology, Johns Hopkins University (S.A.G., R.R., C.H., K.C.S., C.R., J.O.B., D.O.K., P.S., T.P.); Food and Drug Administration (FDA), Silver Spring, Maryland, USA (A.B., E.D., E.W., L.S.L.P., T.P.); Department of Pharmaceutics, University of Minnesota, Minneapolis, Minnesota, USA (W.F.E.); Wake Forest School of Medicine, Winston-Salem, North Carolina, USA (R.E.S., G.L.); Department of Neurosurgery, Barrow’s Neurologic Institute, Phoenix, Arizona, USA (N.S.); Medical Oncology, City of Hope, Duarte, California, USA (J.P.); Department of Neurology, University of Alabama Birmingham, Birmingham, Alabama, USA (B.N.); Department of Radiation Oncology, Mayo Clinic, Rochester, Minnesota, USA (J.N.S.); Department of Medicine, Cleveland Clinic, Cleveland, Ohio, USA (D.P.); Department of Neurology, University of Virginia, Charlottesville, Virginia, USA (D.S.); Department of Oncology, Johns Hopkins University (S.A.G., R.R., C.H., K.C.S., C.R., J.O.B., D.O.K., P.S., T.P.); Food and Drug Administration (FDA), Silver Spring, Maryland, USA (A.B., E.D., E.W., L.S.L.P., T.P.); Department of Pharmaceutics, University of Minnesota, Minneapolis, Minnesota, USA (W.F.E.); Wake Forest School of Medicine, Winston-Salem, North Carolina, USA (R.E.S., G.L.); Department of Neurosurgery, Barrow’s Neurologic Institute, Phoenix, Arizona, USA (N.S.); Medical Oncology, City of Hope, Duarte, California, USA (J.P.); Department of Neurology, University of Alabama Birmingham, Birmingham, Alabama, USA (B.N.); Department of Radiation Oncology, Mayo Clinic, Rochester, Minnesota, USA (J.N.S.); Department of Medicine, Cleveland Clinic, Cleveland, Ohio, USA (D.P.); Department of Neurology, University of Virginia, Charlottesville, Virginia, USA (D.S.); Department of Oncology, Johns Hopkins University (S.A.G., R.R., C.H., K.C.S., C.R., J.O.B., D.O.K., P.S., T.P.); Food and Drug Administration (FDA), Silver Spring, Maryland, USA (A.B., E.D., E.W., L.S.L.P., T.P.); Department of Pharmaceutics, University of Minnesota, Minneapolis, Minnesota, USA (W.F.E.); Wake Forest School of Medicine, Winston-Salem, North Carolina, USA (R.E.S., G.L.); Department of Neurosurgery, Barrow’s Neurologic Institute, Phoenix, Arizona, USA (N.S.); Medical Oncology, City of Hope, Duarte, California, USA (J.P.); Department of Neurology, University of Alabama Birmingham, Birmingham, Alabama, USA (B.N.); Department of Radiation Oncology, Mayo Clinic, Rochester, Minnesota, USA (J.N.S.); Department of Medicine, Cleveland Clinic, Cleveland, Ohio, USA (D.P.); Department of Neurology, University of Virginia, Charlottesville, Virginia, USA (D.S.); Department of Oncology, Johns Hopkins University (S.A.G., R.R., C.H., K.C.S., C.R., J.O.B., D.O.K., P.S., T.P.); Food and Drug Administration (FDA), Silver Spring, Maryland, USA (A.B., E.D., E.W., L.S.L.P., T.P.); Department of Pharmaceutics, University of Minnesota, Minneapolis, Minnesota, USA (W.F.E.); Wake Forest School of Medicine, Winston-Salem, North Carolina, USA (R.E.S., G.L.); Department of Neurosurgery, Barrow’s Neurologic Institute, Phoenix, Arizona, USA (N.S.); Medical Oncology, City of Hope, Duarte, California, USA (J.P.); Department of Neurology, University of Alabama Birmingham, Birmingham, Alabama, USA (B.N.); Department of Radiation Oncology, Mayo Clinic, Rochester, Minnesota, USA (J.N.S.); Department of Medicine, Cleveland Clinic, Cleveland, Ohio, USA (D.P.); Department of Neurology, University of Virginia, Charlottesville, Virginia, USA (D.S.); Department of Oncology, Johns Hopkins University (S.A.G., R.R., C.H., K.C.S., C.R., J.O.B., D.O.K., P.S., T.P.); Food and Drug Administration (FDA), Silver Spring, Maryland, USA (A.B., E.D., E.W., L.S.L.P., T.P.); Department of Pharmaceutics, University of Minnesota, Minneapolis, Minnesota, USA (W.F.E.); Wake Forest School of Medicine, Winston-Salem, North Carolina, USA (R.E.S., G.L.); Department of Neurosurgery, Barrow’s Neurologic Institute, Phoenix, Arizona, USA (N.S.); Medical Oncology, City of Hope, Duarte, California, USA (J.P.); Department of Neurology, University of Alabama Birmingham, Birmingham, Alabama, USA (B.N.); Department of Radiation Oncology, Mayo Clinic, Rochester, Minnesota, USA (J.N.S.); Department of Medicine, Cleveland Clinic, Cleveland, Ohio, USA (D.P.); Department of Neurology, University of Virginia, Charlottesville, Virginia, USA (D.S.); Department of Oncology, Johns Hopkins University (S.A.G., R.R., C.H., K.C.S., C.R., J.O.B., D.O.K., P.S., T.P.); Food and Drug Administration (FDA), Silver Spring, Maryland, USA (A.B., E.D., E.W., L.S.L.P., T.P.); Department of Pharmaceutics, University of Minnesota, Minneapolis, Minnesota, USA (W.F.E.); Wake Forest School of Medicine, Winston-Salem, North Carolina, USA (R.E.S., G.L.); Department of Neurosurgery, Barrow’s Neurologic Institute, Phoenix, Arizona, USA (N.S.); Medical Oncology, City of Hope, Duarte, California, USA (J.P.); Department of Neurology, University of Alabama Birmingham, Birmingham, Alabama, USA (B.N.); Department of Radiation Oncology, Mayo Clinic, Rochester, Minnesota, USA (J.N.S.); Department of Medicine, Cleveland Clinic, Cleveland, Ohio, USA (D.P.); Department of Neurology, University of Virginia, Charlottesville, Virginia, USA (D.S.); Department of Oncology, Johns Hopkins University (S.A.G., R.R., C.H., K.C.S., C.R., J.O.B., D.O.K., P.S., T.P.); Food and Drug Administration (FDA), Silver Spring, Maryland, USA (A.B., E.D., E.W., L.S.L.P., T.P.); Department of Pharmaceutics, University of Minnesota, Minneapolis, Minnesota, USA (W.F.E.); Wake Forest School of Medicine, Winston-Salem, North Carolina, USA (R.E.S., G.L.); Department of Neurosurgery, Barrow’s Neurologic Institute, Phoenix, Arizona, USA (N.S.); Medical Oncology, City of Hope, Duarte, California, USA (J.P.); Department of Neurology, University of Alabama Birmingham, Birmingham, Alabama, USA (B.N.); Department of Radiation Oncology, Mayo Clinic, Rochester, Minnesota, USA (J.N.S.); Department of Medicine, Cleveland Clinic, Cleveland, Ohio, USA (D.P.); Department of Neurology, University of Virginia, Charlottesville, Virginia, USA (D.S.)

**Keywords:** blood-brain barrier, brain cancer, brain permeability, chemotherapy, targeted therapies

## Abstract

Current assessments of brain permeability rely predominantly on drug delivery to contrast-enhancing tumor regions. However, substantial portions of central nervous system (CNS) tumors reside within non-enhancing brain (NEB), where drug concentrations frequently remain subtherapeutic. This collaborative Adult Brain Tumor Consortium and Food and Drug Administration workshop aimed to identify criteria for defining NEB permeability to accomplish 2 critical objectives: (1) allocate clinical trial resources toward agents achieving therapeutic NEB concentrations and (2) minimize systemic toxicity when CNS benefit is improbable. The workshop systematically evaluated permeability assessment modalities, including drug physicochemical properties, in vitro blood-brain barrier models, and penetration into cerebrospinal fluid and normal rodent brain. Methodological approaches to determine requisite NEB drug concentrations and approaches to measuring NEB pharmacokinetics and pharmacodynamics were examined. This culminated in developing the Non-Enhancing Brain Permeability Index (NEBPI), which assigns therapeutic agents to 3 categories: sufficiently permeable, insufficiently permeable, or impermeable. The NEBPI provides a standardized framework to assist investigators and regulatory agencies to evaluate NEB penetration before human efficacy studies are initiated for agents that require direct tumor contact. Assessing NEB drug penetration is critical to improving outcomes in CNS tumors and reducing the incidence of brain metastases in systemic malignancies.

Key Points

- A standardized assessment of drug penetration in non-enhancing brain (NEB) is needed to objectively evaluate and define NEB permeability.
- Selection of NEB-permeable drugs aims to reduce the incidence of brain metastases and improve brain tumor outcomes.

## Background

There is currently no consensus on what defines a “brain permeable” drug in central nervous system (CNS) oncology. Novel agents for CNS cancers are frequently deemed “brain permeable” based on physicochemical properties, preclinical models, or perioperative studies where drug concentrations are measured in resected contrast-enhancing tumor tissue. However, given the extensive infiltration of CNS cancers into normal brain, successful targeting of infiltrative tumor in the non-enhancing brain (NEB) is required to effectively treat CNS tumors or to reduce the incidence of brain metastases. As the blood-brain barrier (BBB) excludes the vast majority of systemically administered FDA-approved drugs, reliance on an imprecise definition of “brain permeable” encourages the conduct of efficacy trials that expose patients to systemic toxicities with a low likelihood of meaningful benefit in non-enhancing CNS disease.

**Figure 1.**
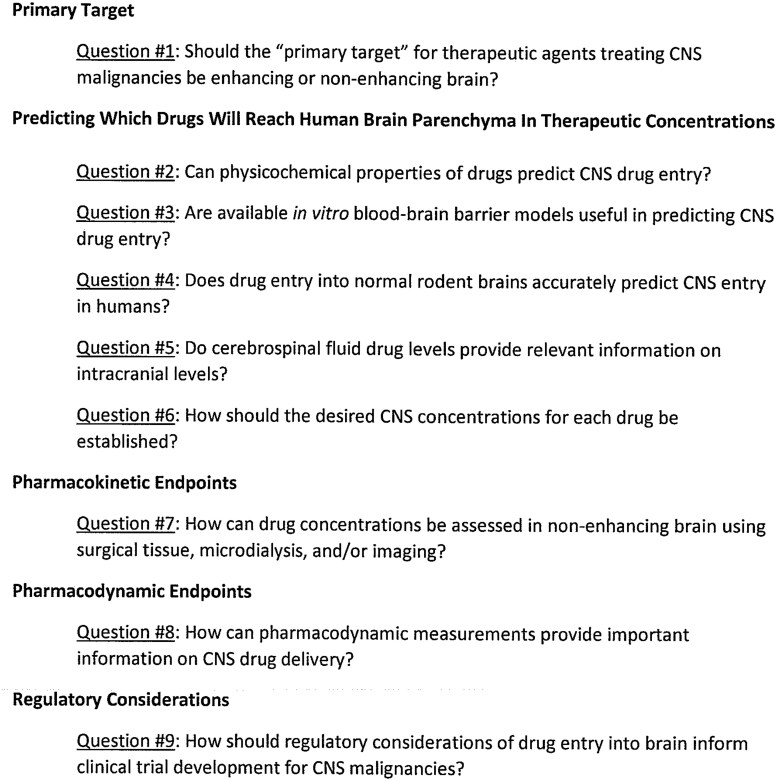
Primary questions addressed at this workshop.

To address the absence of standardized criteria defining a “brain permeable” agent, investigators from the Adult Brain Tumor Consortium (ABTC), a National Cancer Institute funded phase I/II clinical trial group, and the Food and Drug Administration (FDA) convened a multidisciplinary expert panel. These clinicians, preclinical and clinical investigators, clinical pharmacologists, imaging researchers, and regulatory scientists participated in an in-person workshop in Baltimore, Maryland, on May 20, 2024. The primary objective was to establish evidence-based criteria for evaluating the BBB permeability of systemically administered drugs prior to initiation of efficacy trials in patients with brain tumors. The specific questions addressed are shown in Figure 1. This manuscript provides expert consensus responses to these critical questions, methodologies for assessing penetration of therapeutic agents into non-enhancing CNS regions, and research priorities to enhance therapeutic success.

Despite the remarkable recent advances in cancer treatment, the overall mortality rates for primary brain tumors have remained essentially unchanged for thirty years.^1^ Since 2000, temozolomide has been the only drug approved by the FDA for the treatment of newly diagnosed glioblastoma, the most common and deadly adult primary brain tumor. When combined with standard radiation, concurrent and adjuvant temozolomide extends survival from 12 to 14.6 months.^2^ This benefit is largely restricted to the 40% of patients with MGMT methylated tumors and is not associated with curative potential.^3^ Clinical trials investigating diverse cytotoxic regimens, targeted therapies, anti-angiogenic agents, and immunotherapy approaches have yielded disappointing results.^4^

A critical difference between CNS and systemic malignancies is the presence of the BBB. This evolutionarily conserved protective barrier maintains nervous system homeostasis, improves the efficiency and reliability of neural signaling, regulates molecular transport systems, and restricts CNS entry of pathogens and toxins.^5^ The human BBB excludes virtually all large molecular weight compounds and the majority of FDA-approved drugs, creating a formidable obstacle to drug development.^6^ Glioblastomas harboring actionable mutations often fail to respond to targeted agents effective in many extracranial cancers with similar mutational profiles.^7^ Similarly, patients with widely metastatic solid tumors that are responding to systemically administered targeted therapies often experience a first site of progression in brain where drug concentrations are insufficient.^8^ Various approaches have been explored to improve CNS drug delivery, including intraventricular administration, direct parenchymal injection, convection-enhanced delivery, placement of drug-eluting implants, intra-arterial chemotherapy, and transient BBB disruption via intra-arterial mannitol or focused ultrasound.^9^ However, to date, these local interventions have had limited impact on improving survival in patients with brain tumors or preventing brain metastasis.

Given that drug efficacy usually requires therapeutic concentrations in target tissues, BBB permeability represents a critical factor in selecting promising agents for CNS malignancies.^10^ Currently, no formal definition of a “brain permeable drug” exists, creating uncertainty in drug development and clinical decision-making. This workshop represented a collaborative effort by the National Institutes of Health funded ABTC and the FDA to explore expert consensus-based criteria. The initiative addresses critical questions designed to inform preclinical and clinical investigators, industry, institutional review boards, and regulatory agencies by standardizing CNS permeability terminology and elucidating the value and limitations of such definitions.

This workshop specifically addressed brain permeability requirements for agents requiring direct tumor penetration for therapeutic effect and how to best document and quantify delivery of systemically administered agents in NEB tumor tissue. As a result, this discussion may be less relevant for: (1) enhancing primary brain tumors or brain metastases where systemically administered drugs are more likely to gain access to tumor tissue or (2) for technologies, agents or approaches where local drug concentrations in the brain are less important, such as immunotherapies that activate systemic lymphocytes that travel to the brain or anti-angiogenesis drugs that primarily target vasculature rather than intraparenchymal tumor. Specific drug delivery issues in leptomeningeal or intraocular disease were considered outside the scope of this workshop.

## Selection of the Appropriate Tissue to Target

### Question #1: Should the “Primary Target” for Therapeutic Agents Be Enhancing or Non-Enhancing Brain?

#### Primary brain tumors

Magnetic resonance imaging (MRI) provides detailed ­visualization of both tumor and peritumoral edema on T2-weighted and FLAIR sequences. More extensive disruption of the BBB allows gadolinium-based contrast agents (MW 500-800) to penetrate the tumor at concentrations visible on MRI. In lower-grade tumors, imaging abnormalities are often confined to FLAIR hyperintensity without contrast enhancement while higher-grade tumors are typically characterized by enhancing tumor surrounded by FLAIR signal abnormality. However, histopathological studies have clearly demonstrated that both low- and high-grade gliomas infiltrate well beyond the regions of FLAIR abnormality.^11^^,^^12^ As a result, standard radiation fields designed to encompass the entire tumor volume include the FLAIR volume plus a margin of normal-appearing brain.^13^ At the time of original presentation, only 20% of the total FLAIR volume of a glioblastoma enhances with gadolinium.^14^ Not surprisingly, neurosurgical efforts to resect all contrast-enhancing tumor have not resulted in cure and provide only modest survival benefit.^15^ As a result, the non-enhancing tumor volume must be considered the principal therapeutic target for systemically administered therapies requiring direct tumor exposure. Similarly, in low-grade gliomas the primary target of interest is NEB as this is the bulk of the tumor volume.

#### Brain metastases

Brain metastases are approximately 10 times more common than primary brain tumors. They often present with neurological signs and symptoms and one or more contrast-enhancing lesions on MRI that are generally well-circumscribed and can be treated with combinations of surgery, radiation, and other locally directed therapies.^16^ Systemically administered drugs reach these enhancing lesions to varying degrees and can result in clinical and radiographic responses. However, many patients harbor micro-metastatic lesions that are not yet visible on MRI and these micro-metastases can only be reached by agents that penetrate a normal BBB. In addition, there are increasing reports of patients with metastatic cancers on targeted agents that develop brain metastases as the first site of progression or relapse due to subtherapeutic CNS drug levels.^17^^,^^18^ Efforts to reduce the incidence of brain metastases, whether in the adjuvant or metastatic setting, will rely on agents able to penetrate a relatively normal BBB as contrast enhancement is not evident until a sizeable metastatic lesion is established.

## Predicting Which Drugs Will Reach Human Brain Parenchyma in Therapeutic Concentrations

Systemically administered CNS-directed agents should be selected based on their ability to reach the target tissue in enhancing and NEB parenchyma in therapeutic concentrations. Many factors have been considered to estimate the likelihood that an agent will meet this goal, including: (1) physicochemical properties, (2) in vitro BBB models, (3) in vivo preclinical models, and (4) distribution into cerebrospinal fluid (CSF). Each is discussed below.

### Question #2: How Predictive Are Physicochemical Properties (ie, Size, Lipophilicity, PKA, Efflux Liability, etc.) for Drug Entry and Retention in the Brain?

**Figure 2.**
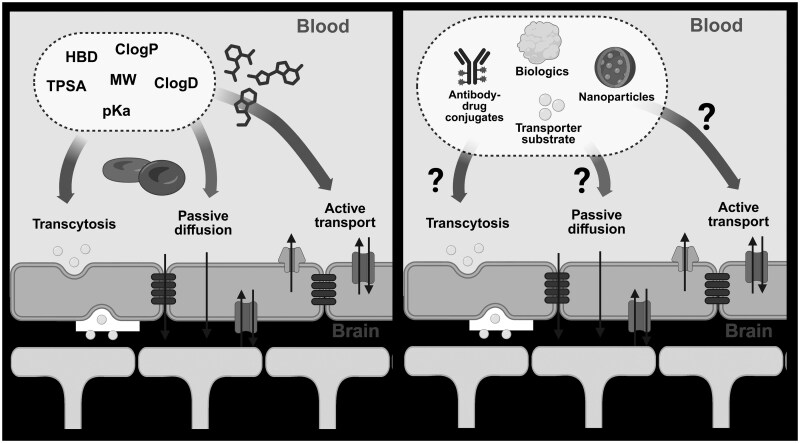
General parameters used to predict BBB permeability and factors limiting permeability. Left panel: Parameters used to predict BBB permeability. Right panel: Factors limiting permeability.

Physical and structural characteristics of a compound that enable transport are related to anatomical and biochemical features of the BBB (see Figure 2). As a rule, small, moderately lipophilic molecules (CLogP ∼2.5) with a molecular weight below 400 Daltons cross the BBB through passive transcellular diffusion.^19^ In contrast, polar or charged molecules generally exhibit poor brain penetration and depend on active transport mechanisms for effective delivery. Large molecules, such as proteins and antibodies, require receptor-mediated endocytosis to enter the CNS. Additional factors affecting CNS penetration include hydrogen bonding, ionization properties, and molecular flexibility.

Following the seminal study by Lipinski outlining the criteria for oral bioavailability, known as the “Rule of 5”, similar algorithms (beyond lipophilicity and charge) using physicochemical properties have attempted to enhance the predictability of CNS penetrance of drug molecules.^20^ The composite score by Wager and colleagues, known as CNS “multiparameter optimization” (MPO), focuses on 6 fundamental physicochemical properties: ClogP; distribution coefficient at pH = 7.4 (ClogD); topological polar surface area, number of hydrogen bond donors, molecular weight and ionization constant of the most basic center.^21^ While these descriptors provide a viable benchmark for designing new chemical entities, the dataset used to derive the CNS MPO includes enhancing CNS targets and was validated using preclinical models that are known to deviate significantly from human physiology. Hence, although CNS MPO is a useful metric in designing molecules, it is essential to corroborate in silico predictions with in vitro and in vivo assays to make more informed predictions regarding individual molecules’ ability to reach brain tissue targets.^22^

### Question #3: Are Currently Available in Vitro Blood-Brain Barrier Models Useful in Predicting CNS Drug Entry?

In vitro models of the BBB span a wide range of complexity from high throughput 2D microfluidic models to more complex tissue-engineered systems.^23^^,^^24^ The first tissue-engineered BBB models were developed around 10 years ago taking advantage of advances in stem cell technology to differentiate human brain microvascular endothelial cells that exhibit physiological barrier function.^25^^,^^26^ It is now possible to produce complex multicellular models of microvessels and capillaries in an extracellular matrix under shear flow. Recognizing the important role of supporting cells on BBB function led to the concept of the neurovascular unit (NVU) and the incorporation of pericytes and astrocytes into these models.^25^^,^^27^ Although complex vascular models of the tumor microenvironment have been demonstrated, this capability has yet to result in a meaningful impact on clinical neuro-oncology.^28^ In the future, increasingly sophisticated BBB models can be used to enhance high-throughput screening for agents that cross the BBB, provide mechanistic insight on drug transport, and provide valuable information on novel approaches for chemical (eg, hyperosmotic mannitol) or mechanical (eg, MB-focused ultrasound) BBB opening to increase CNS drug delivery.

### Question #4: Does Drug Entry into Normal Rodent Brains Accurately Predict CNS Entry in Humans?

The functional barrier between systemic circulation and brain is highly conserved across evolution. In vertebrate animals with a circulatory system, the BBB shares significant commonalities from zebrafish to humans with continuous endothelial tight junctions supported by an NVU. While more primitive than in mammals, the NVU in zebrafish includes endothelial cells, pericytes, and glial cells. The density of astrocytic end-feet is higher in humans than mice, but otherwise the NVU is highly similar.^29^ At a molecular level, zebrafish and murine endothelial cells share numerous functionalities including expression of homologous proteins involved in maintaining tight junctions (ZO-1, Cldn5), glucose transport (Glut1), suppressor of transcytosis (Mfsd2a), and xenobiotic efflux (P-gp, BCRP) although it is not yet clear how these differences impact BBB function.^30^ Given these commonalities across vertebrates, both zebrafish and mice are useful models to study various aspects of the BBB.

Mice are a frequently used translational model for predicting drug distribution across the BBB in humans. The relevance of murine models in studying human physiology and pharmacology is well established.^22^ Of course, there are salient differences between mice and humans that need to be addressed. In general, the pharmacokinetic (PK) profiles of most small molecule drugs vary between mice and humans, with the disposition of many molecules being much faster in mice. These differences in systemic PK translate to differential PK of drug exposure in the CNS compartment. The orders of magnitude larger size of the brain and relative size of distinct brain regions also must be considered when evaluating the translational potential of in vivo models.^31^ As one example, the rodent olfactory bulb is, relatively, much larger than in humans and distances to other regions of the brain are much smaller. These differences may have an important impact on the human translation of nasal delivery strategies developed in rodents.

Comparisons of gene and protein expression within brain capillary endothelial cells in mice and humans are also well documented. RNA sequencing and proteomic studies have demonstrated differential expression levels for numerous solute carriers and ABC transporters, including higher expression of Pgp and lower expression of BCRP in mice.^32–34^  While the functional effect of varying efflux transporter expression is specific to each drug, many drugs show comparable levels of CNS restriction in both mice and humans.^35^^,^^36^ Similar capabilities of the rodent and human BBB are not surprising, but because of species-specific differences in potential drug transporters and individual drug PK behavior, measurements of brain permeability in rodents are not always predictive for humans.^37^^,^^38^ Nevertheless, studies in rodents can be an instructive step towards determining if a candidate drug will reach therapeutic concentrations in human brain.

### Question #5: Do Cerebrospinal Fluid Drug Levels Provide Relevant Information on Intracranial Levels?

The relative ease and safety of repeatedly sampling CSF encourages the use of CSF levels to serve as a surrogate for drug concentrations within the brain. However, significant differences exist between the BBB and the blood-CSF barrier (BCB). The BBB is composed of perivascular endothelial cells connected by tight junctions, whereas the BCB is composed of the epithelial plasma membrane of the choroid plexus with fenestrations.^39^ As the BCB is inherently more permeable than the BBB drugs that reach detectable concentrations in the CSF may not cross an intact BBB. This is especially true for water-soluble molecules. Multiple clinical studies have consistently demonstrated significant disparities between brain and CSF total and unbound drug levels.^40–42^ Beyond the structural differences governing access to these compartments, there are also methodological challenges related to microscopic blood contamination of accessed CSF. As a result, direct measurement of drug levels in the CSF is not considered a reliable surrogate for drug levels within the brain parenchyma or CNS tumor tissue.

## Pharmacokinetic Endpoints

### Question #6: How Should a “Target PK Concentration” for a Drug Be Established Prior to Initiating Clinical Efficacy Trials?

**Figure 3.**
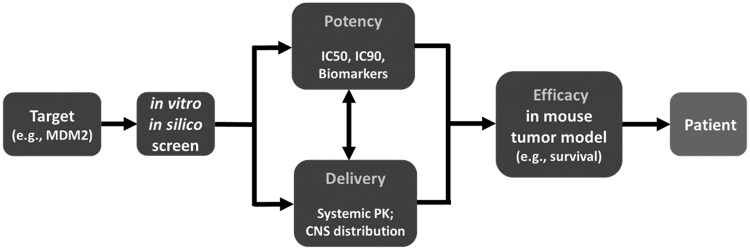
Delivery-potency-efficacy paradigm for the development of drugs for CNS diseases. As the development process flows from target validation to early clinical trials, several questions must be answered along the way. Not the least of these are determining: (1) How much is enough? (potency) and (2) Can one achieve that concentration in the tumor? (delivery). Key to the ultimate success of this process is a consideration of both potency and delivery as contributing factors for the preclinical in vivo efficacy of a compound, and its eventual promotion for use in clinical trials.

A key unanswered question is what concentration is required, for how long, and to what locations, to yield a favorable risk: benefit profile in the CNS. This depends on the potency and toxicity profile of the agent. In essence, this is related to a therapeutic window whose limits are typically characterized by the systemic toxicity profile, maximum tolerated plasma concentration (dose) and the minimum efficacious concentration in the tumor. One straightforward way to estimate a target concentration would include determining the IC90 of the drug in an in vitro assay, measuring the unbound drug in the in vitro media, and hence determining a target unbound tumor concentration.^43^^,^^44^ The ability to achieve the minimum efficacious concentration in the brain and tumor depends on the brain penetrability and the potency of the drug. In the drug development paradigm, potency and permeability need to be evaluated together (Figure 3), since an extremely potent compound may not need robust BBB permeability to be efficacious. Taken to its extreme, a compound that has a brain or tumor partition coefficient of only 0.01 (ie, “one percent brain penetration”), may be efficacious if that level of permeability results in an efficacious and tolerable concentration. Therefore, both permeability and potency need to be considered together in any screening process as an extremely potent drug may be efficacious even with a low level of penetration.^45^

One example of this is the preclinical development of brigimadlin, a potent MDM2 inhibitor.^46^ Brigimadlin is a novel MDM2-p53 antagonist with robust preclinical activity in glioblastoma models. The contributions of drug potency and CNS delivery to the in vivo efficacy of brigimadlin have been evaluated in patient-derived xenograft models of glioblastoma. The excellent preclinical efficacy of brigimadlin in glioblastoma is primarily a result of remarkable potency even though its CNS delivery is significantly limited by BBB efflux. These findings reaffirm an often-lost translational perspective on drug development, that is, the “Delivery—Potency—Efficacy” relationship (see Figure 3).

Other key concepts that deserve consideration include: (1) Given the invasive nature of many brain tumors, what is the specific location where the target concentration is needed and what is the condition of the BBB at those locations? (2) Should we be looking at an unbound concentrations as the pharmacologically relevant endpoint? (3) What pharmacological metrics should be considered for correspondence with a pharmacodynamic (PD) endpoint, either a biomarker or efficacy, (4) How will the target concentration at a specific location be affected by combination therapies, including radiation, (5) At what stage of drug development would this target concentration be relevant?, and (6) How will possible interspecies differences between animal models and patients be evaluated?

### Question #7: How Can Drug Concentrations Be Assessed in Human Non-Enhancing Tumor?

Once a drug has been selected and the target minimally effective concentration has been determined, the next step is to ensure that it reaches the desired concentration in NEB in humans. This may include PK or PD endpoints. The PK endpoints generally require tissue biopsies, microdialysis, or imaging studies.

#### Surgically resected tissue

Tissue acquisition and analysis are essential elements in brain tumor PK/PD-driven clinical studies. Historically, the total brain-to-plasma concentration ratio uncorrected for residual blood in the tissue has been reported in the literature as a measure of drug-brain penetration. The Free-Drug Hypothesis, however, suggests that total drug concentration ratios have limited generalizability, as nonspecific binding of a drug to proteins and lipids in plasma and the brain leads to an overestimation of expected drug performance. Because drug binding is especially different in cell culture compared to tissues, evaluation of free drug concentrations is important when using in vitro studies to define a minimally effective concentration. Unbound drug concentrations drive in vivo pharmacological effects, and thus the use of the unbound brain-to-plasma concentration ratio is essential to measure brain tumor drug penetrance. Total and unbound drug concentrations in plasma and tumor tissues should be measured in each PK/PD-driven clinical study, typically using an equilibrium dialysis method combined with LC-MS/MS analysis.

Tumor tissue selection and acquisition play an important role in tissue-based studies seeking to understand PK performance. The BBB is heterogeneously disrupted within most intracranial tumors and especially in high-grade gliomas. Regions of gadolinium-enhancing and non-enhancing tumors have substantially different BBB properties. Moreover, it has been shown that significant heterogeneity in BBB permeability exists between and within glioblastomas using highly permeable (levetiracetam) and nonpermeable (cefazolin) agents as markers of BBB permeability.^47^ Of note, it has been shown to be critical to correct tissue concentrations for the amount of drug “contamination” by the residual blood in the specimen: this is especially true for compounds with limited BBB permeability.^47^ Given the nature of surgical sampling, each specimen may have very different amounts of drug contributed by a variable contribution by residual blood, and therefore to get truly informative data on drug BBB penetration, the residual blood in each specimen needs to be determined. A useful method to do this, that employs measuring hemoglobin in the resected specimen as a marker of residual blood, has recently been published.^47^ Additionally, in the setting of recurrent disease, regions of progression versus pseudoprogression can be intermixed within a single mass and each of these compartments can harbor variable levels of unbound drug concentrations. To optimize tissue PK analyses, tissue acquisition for unbound drug measurements should sample across these compartments, but focus on non-enhancing tumor regions, where the BBB is comparatively intact, avoiding the risk of overestimating drug penetration. The integration of physiological imaging paradigms, such as preoperative perfusion MR imaging, can further refine regions of interest in newly diagnosed and recurrent disease.

#### Cerebral microdialysis

This sampling technique enables quantification of free drug concentrations in brain extracellular fluid (ECF) over time. Cerebral microdialysis is FDA-approved for use in individuals with traumatic brain injury to follow serial lactate levels in a particular location in the brain, but it has also been applied in early drug development of potential brain tumor therapies. These small microdialysis catheters can be placed in brain tumor patients at the time of surgical debulking in regions of either enhancing or NEB tissue on MRI. Microdialysis sampling has been utilized in both preclinical and clinical studies as a tool to assess the pathophysiology, pharmacokinetics, and pharmacodynamics of chemotherapy agents.^48–52^ Continuously measuring free drug concentrations of a chemotherapy in brain ECF with microdialysis catheters can provide more accurate information about how “blood-brain barrier penetrant” a drug is compared to studies in animal models or in humans through the analysis of brain homogenates, which tend to overestimate BBB penetration and provide only a single measurement in time.

Although cerebral microdialysis has advantages over other techniques used to measure drug concentrations in brain parenchyma, it also has some limitations. Many anti-cancer agents cannot be efficiently dialyzed due to their chemical properties (high molecular weight, aqueous insolubility, and lipophilicity).  The technique is intensive in terms of time and cost because it must be performed in the inpatient setting. Additionally, the drug measurements are from a very small region around the catheter. To accurately determine ECF concentrations of a drug, it is first necessary to conduct studies to estimate the compound’s in vivo fractional recovery. Several methodologies to assess this have been proposed.^53^^,^^54^

#### Pharmacokinetic imaging

Pharmacokinetic imaging can provide unique insights into in vivo drug distribution within the human brain, including regions of non-enhancing tumor. This non-invasive approach is particularly appealing when invasive alternatives are limited, such as studying the normal-appearing brain.^55^ Brain permeability of drugs can be visualized through leveraging PK and PD parameters.^56^^,^^57^ Ideal techniques should provide temporally dynamic data with sufficient resolution to discriminate underlying tissue types (eg, white matter, gray matter, tumor). While expensive and niche techniques may provide valuable PK/PD data for window of opportunity surgical studies, the development of cost-effective solutions that require no specialized equipment or localized expertise is crucial.^58–60^ This will facilitate the goal of using imaging for patient enrichment in clinical trials and personalized treatment planning.

Pharmacokinetic imaging exploits the molecular or physicochemical properties of drugs that distinguish them from surrounding tissue. Radiolabeling isotopes attached to drugs for positron emission tomography (PET) is the most widely recognized approach for PK imaging. PET is extremely sensitive, with an ability to detect drug (radioactivity) concentration changes in the picomolar range.^60^ The spatial resolution limitations of PET can be mitigated through co-registration with structural MRI sequences.^60^ However, PET faces numerous feasibility barriers, including the radiolabeling process (eg, [^11^C]methylation) changing the PK of the original drug, or the high costs associated with radiochemistry and cyclotron facilities, as well as the required regulatory reviews.^60^ Microdosing strategies, which use 1/100th of the proposed effective dose, can mitigate some of these concerns while providing valuable information.

## Pharmacodynamic Endpoints

### Question #8: Can Pharmacodynamic Measurements Provide Important Information on CNS Drug Delivery?

Pharmacodynamic endpoints are critically important to fully understand target engagement and mechanism of action and to guide clinical strategies. In CNS cancers, there are many situations in which obtaining a drug level within the target tissue is difficult or impossible—in those cases PD endpoints can be useful surrogate indicators of drug access to the target of interest.

For questions related to drug efficacy, PD endpoints should be relevant to the mechanism of action (if possible) and proximal to the efficacy endpoint. These can be useful to inform go/no-go decisions regarding further development. In the case of the PI3K inhibitor, buparlisib, researchers were able to demonstrate drug entry into the brain and modest decreases in the dosing PD biomarker phospho-AKT but no suppression of downstream response PD endpoints (phospho-S6 and proliferation Ki-67) or improvement in survival.^61^^,^^62^ Imaging-based PD biomarkers, such as 2-hydroxygluterate levels in IDH-mutant astrocytoma or beta-hydroxybutyrate levels following a ketogenic diet intervention, can also be useful non-invasive indicators of drug entry into the target tissue.^63^^,^^64^ Notably, it is challenging to identify PD endpoints that are useful to assess both drug access to the site-of-action as well as potential ­efficacy at the same time point for tissue acquisition, and a staggered window of opportunity surgical study design may be necessary to obtain both.^65^

While PD endpoints can be useful surrogates for drug engagement at the target tissue, there are significant barriers to implementation. First, most PD endpoints are not FDA-qualified and assays lack standardization and rigor. Second, the biological variability of a given PD endpoint needs to be characterized in order to evaluate the potential effect of the treatment and determine sample size—this includes inter-patient variability, intra-tumoral heterogeneity and change over time. Third, assay-specific parameters add a layer of complexity and potential error to trial design and successful completion (time to tissue preservation, image acquisition location, stability of pathway inhibition, etc.).^66^ Despite these concerns, the power of PD endpoints to demonstrate drug access/effect on target tissue, and to establish a dose-response relationship, make them powerful components of early-phase drug development. However, to use a PD biomarker as a primary endpoint, the FDA recommends requesting a meeting early in development and prior to initiation of any trial intended to support registration.

#### Pharmacodynamic imaging

Pharmacodynamic imaging broadens the scope of techniques to confirm drug effects and by extension, drug distribution. It offers potential readouts in both tumoral and normal-appearing brain tissue and may be particularly useful in dose-finding studies for experimental drugs.^57^^, ^^67^^, ^^68^ PD imaging might leverage changes in metabolic activity, such as glucose uptake. For example, reductions in gray matter glucose metabolism in response to drugs targeting EGFR or its downstream pathway can confirm BBB permeability in the normal-appearing brain and complement observations in tumoral regions.^57^ Additionally, amino acid PET tracers such as [^18^F]fluoro-ethyl-tyrosine and others show elevated uptake in most active tumors, often outside the enhancing area, and thus may provide a better representation of metabolic drug effects.^69^^,^^70^ Dynamic contrast-enhanced perfusion MRI may enable detection of perfusion changes related to drug therapy.^71^ Magnetic resonance spectroscopy can concurrently detect metabolic reporters such as lactate, which reflects Warburg metabolism and hypoxia within the tumor, or glutamate which may offer insights into both tumor and normal-appearing brain.^70^^,^^72^ This can be particularly useful to study metabolic modulator drugs, such as dichloroacetate.^68^ A particularly relevant application is monitoring tissue 2-hydroxyglutarate in response to IDH inhibitor therapy in IDH-mutated gliomas.^73^ Chemical exchange saturation transfer (CEST) imaging further expands the range of observable PD parameters, such as pH changes induced by treatment.^72^^,^^74^ An important limitation currently facing PD imaging is its specificity to experimental drug effect(s), as common imaging readouts are often confounded by epiphenomenological events associated with the pathogenesis of CNS tumors and/or their accompanied treatments. For example, the 2-HG reductions observed on MRI have yet to be linked to clinical efficacy of IDH inhibitor therapy.^75–77^

## Regulatory Considerations

### Question #9: How Does the FDA Currently Evaluate “Brain Permeability” When Considering Drug Trials for Patients with Primary and Metastatic Brain Tumors?

When assessing “brain permeability” of an investigational drug to treat CNS tumors, FDA considers nonclinical and clinical evidence that the drug penetrates the brain with an intact BBB, achieves likely therapeutic concentrations, and persists long enough to have the desired effects.

Nonclinical data submitted to FDA to evaluate brain penetration typically include proof-of-concept (PoC) in vitro studies and in vitro and in vivo ADME/PK studies as well as in vivo xenograft studies in rodents. Nonclinical PoC studies conducted to support a dose-escalation trial in patients with CNS tumors provide initial information on drug permeability. FDA nonclinical oncology guidances do not currently recommend specific brain penetration studies, because flexibility is needed due to product diversity and clinical indications.^78^^,^^79^  Thus, the extent of nonclinical brain penetration data submitted to the FDA may be highly variable.

Additional clinical data may include rate of transport into healthy brain, efflux ratio, quantitative product concentration in the brain or CSF relative to plasma, and brain concentration of the drug.^55^ While brain and CSF PK data are rare in early-stage clinical studies, systemic PK data may offer useful insights. Even within a given drug class, CNS product activity may vary, making prospective study of these parameters essential. Relationships between systemic PK and CNS activity may be further complicated by drug-drug interactions, as well as by the formation of active or inactive metabolites with variable activity in the brain, and contamination of the tissue sample by blood. Therefore, FDA also considers other therapies that could impact CNS activity or safety via alteration of PK and/or PD, for example, current or prior radiation therapy, anti-seizure medications, enzyme or transporter modulators, or steroids.

FDA attempts to leverage emerging clinical data to ensure that eligibility criteria, safety monitoring, and dosage selection are appropriate. Study designs that include dosage randomization could be supportive of attributing drug effect to observed changes on imaging, PD markers or adverse events increasing confidence in prioritization of the drug for further development.^80^ Use of modeling approaches can help synthesize and evaluate the data to support dose selection. These models may include exposure-response models (E-R), PK/PD models, population PK models, or population-based PK models. Exposure/response and exposure/toxicity in the CNS are important to ensure a favorable therapeutic index. For example, a drug may enter and remain in the CNS compartment at high concentrations, but if it also lowers the seizure threshold or increases the risk of intracranial hemorrhage, the toxicity may outweigh any potential benefit of anti-tumor activity. A major limitation of these modeling approaches is the lack of human samples to validate the models. In these cases, any assumptions used in the models must be justified with available supportive data and may impact the reliability and interpretability of the final model. CNS penetrance should be considered and prospectively evaluated for novel therapeutics when feasible. Sponsors are encouraged to contact FDA early and often throughout the development of drugs intended to treat CNS tumors or metastases.^81^

## Discussion

The BBB is extraordinarily efficient at preventing pathogens and potentially toxic agents in the blood from gaining access to the CNS. This presents unique challenges in treating primary brain tumors and reducing the incidence of brain metastases. MRI scans readily identify regions of the brain where the BBB is sufficiently disrupted to allow gadolinium to penetrate brain parenchyma. However, this contrast agent has a low molecular weight, is not an efflux pump substrate, and is detected on MRI at low concentrations. While contrast-enhancing areas highlight the most permeable regions in the brain, chemotherapy and targeted agents are often large, charged, efflux pump substrates and must reach therapeutic concentrations at the tumor to be effective. In addition, both high- and low-grade primary brain tumors exist in areas of the brain where there is no contrast enhancement. Recent data suggests that only 20% of the total FLAIR volume of newly diagnosed glioblastomas enhance with contrast.^14^ Thus, even if therapeutic agents are able to penetrate and effectively treat the contrast-enhancing regions, 80% of the tumor volume would have minimal exposure to most systemically administered therapeutic agents. While it has yet to be proven that increased drug exposure in non-enhancing tumor will be sufficient to improve outcomes in brain tumors, this question will only be answered once we have delivered therapeutic concentrations of effective agents to non-enhancing tumor. Similarly, in patients with systemic cancers, a drug that is unable to penetrate an intact BBB is unlikely to reduce the incidence of brain metastases. As a result, therapeutic agents that require direct contact with cancer cells to be effective must achieve therapeutic concentrations in NEB to meaningfully improve patient outcomes.

Currently, most agents described as “brain permeable” achieved this designation based on physiochemical properties, preclinical models, or studies where drug concentrations were measured in resected contrast-enhancing tumor specimens. This collaborative ABTC/FDA workshop sought to critically evaluate available information and to assess the screening and prioritization of drugs to treat CNS malignancies. The most important criteria were determined to be: (1) physicochemical characteristics of the drug, (2) CNS permeability in normal animal brain, (3) known minimum effective concentration (MEC), (4) data demonstrating MEC in NEB, and (5) relevant PD effects in NEB (Table 1). Analysis of the physicochemical properties of drugs can identify agents that are unlikely to pass through an intact BBB but are less informative regarding the effect of efflux pumps. PK studies in rodents provide important data on CNS drug permeability through an intact BBB but can be misleading as the systemic metabolism and efflux pumps can be quite different in murine models and humans. Establishing an MEC is routine in the field of infectious disease but not in oncology. However, data are required to set a target dose that one hopes to reach in NEB, and this must consider the potency of the drug against the tumor being treated. Pharmacokinetic and/or PD studies in NEB provide valuable insight into the potential utility of candidate drugs. Less helpful information includes CSF drug levels in animals or humans, radiographic or clinical responses in contrast-enhancing lesions in animals or humans, and permeability data from traditional in vitro BBB models. In summary, a drug that: (1) has physicochemical characteristics that suggest that it should pass the BBB, (2) readily crosses the BBB in normal rodents, (3) has a known MEC that can be used as a target MEC in human PK studies, and (4) reaches the MEC or has relevant PD effects in NEB would be an ideal candidate for efficacy studies in primary brain tumors or prevention studies in CNS metastases.

**Table 1.** Non-enhancing brain permeability index (NEBI) part I: criteria to evaluate the “brain permeability” of systemically administered drugs in non-enhancing brain

Evaluation criteria	Level of importance^a^
Physical characteristics of drug	A
Permeability data in normal animal brain	A
Known minimum effective concentration (MEC—potency)	A
Pharmacokinetic data demonstrating MEC in non-enhancing brain	A
Relevant pharmacodynamic effects in non-enhancing brain	A
CSF drug levels from normal animals or humans	C
Responses in contrast-enhancing lesions in animals or humans	C
Permeability data from in vitro blood-brain barrier models	D

a Levels of importance are graded from A (best) to F (worst).

This overall approach to screening agents for CNS malignancies is encompassed in the Non-Enhancing Brain Permeability Index (NEBPI), which is composed of 2 parts (Tables 1 and 2). Part I of the NEBPI provides a checklist of crucial information that can be used to estimate a drug’s ability to penetrate NEB in therapeutic concentrations. The data that emerge from this are then used in Part II to classify the agents being studied into one of 3 “Brain Permeability Classes.” The first consists of “Sufficiently Permeable” agents. This includes drugs with PK studies in NEB that reach the MEC taking into account the potency of the drug against the targeted tumor, relevant PD effects in NEB, or radiographic responses in NEB. Agents meeting these criteria might be suitable for prevention of CNS metastases and treatment of non-enhancing and enhancing primary brain tumors. Temozolomide would be included in this category as responses have been reported in NEB even though drug levels in the brain are only 20% of those in the blood. “Insufficiently Permeable” agents are those with PK levels below the MEC in NEB with no relevant PD effects or radiographic responses in NEB. These agents might be useful in patients with contrast-enhancing metastases or contrast-enhancing regions of primary brain tumors. “Impermeable” agents have extremely low levels or no drug present in NEB and with no relevant PD effects or radiographic responses in NEB. Theoretically, these agents could be effective in contrast-enhancing metastatic disease or primary brain tumors where the BBB is significantly disrupted. The NEBPI has the potential to be flexible depending upon how the drug is administered, the sensitivity of the tumor to the drug, and the ability of the drug to cross the BBB. For example, methotrexate would be classified as an impermeable agent when it is given to treat glial neoplasms in a standard dose and schedule. However, it would be classified as sufficiently permeable when administered intravenously in high doses to treat primary CNS lymphomas (PCNSL). Even though methotrexate does not easily penetrate the BBB, it is extremely potent against PCNSL and the prolonged and very high blood levels allow sufficient drug in enhancing and NEB to result in radiographic responses and dramatic improvements in survival.^82^

**Table 2.** Non-enhancing brain permeability index (NEBI) Part II: criteria to evaluate the “brain permeability” of systemically administered drugs in non-enhancing brain

Permeability class	Definition	Potential uses
Sufficiently permeable	PK with therapeutic levels in NEB^a^ or Relevant PD effect in NEB or Radiographic responses in NEB	Prevention of CNS metastases Treatment of non-enhancing primary brain tumor Treatment of enhancing tumors
Insufficiently permeable	Low PK levels in NEB^a^ and No relevant PD in NEB and No radiographic response in NEB	Contrast-enhancing metastases Contrast-enhancing primary brain tumor
Impermeable	Little to no drug in NEB^a^ and No relevant PD in NEB and No radiographic response in NEB	Contrast-enhancing metastases Contrast-enhancing primary brain tumor

CNS = central nervous system; NEB = non-enhancing brain; NEBPI = Non-Enhancing Brain Permeability Index; PD = pharmacodynamic; PK = pharmacokinetic.

a Accounting for potency.

This workshop concluded that the NEB is a critical target in the treatment of CNS malignancies and that a standardized approach should be adopted to formally evaluate drug permeability in NEB. NEB permeability data should be provided to investigators, institutional review boards, grant reviewers, and regulatory agencies prior to the initiation of clinical trials in CNS cancers. These steps are designed to ensure that: (1) careful studies of drug penetration into NEB are conducted before efficacy trials are initiated, (2) clinical trial resources are prioritized for the drugs most likely to reach NEB in therapeutic concentrations, and (3) patients with CNS malignancies are not exposed to systemic toxicities from drugs that are very unlikely to provide a significant benefit within the brain. The workshop participants also strongly recommended that future brain tumor research prioritize efforts to develop novel approaches to improve drug delivery to NEB.

## Data Availability

No new data were generated or analyzed in support of this review.
